# Paving the way for future PSMA inhibitors: insights from comparative preclinical evaluations of structure modifications

**DOI:** 10.1186/s41181-025-00389-w

**Published:** 2025-09-26

**Authors:** Katarína Hajduová, Kateřina Dvořáková Bendová, Miloš Petřík, Martina Benešová-Schäfer, Martin Schäfer, Marián Hajdúch, Zbyněk Nový

**Affiliations:** 1 Institute of Molecular and Translational Medicine, Faculty of Medicine and Dentistry, Palacky University Olomouc, Hnevotinska 1333/5, Olomouc, 77900 Czech Republic; 2 Czech Advanced Technology and Research Institute, Palacky University Olomouc, Hnevotinska 1333/5, Olomouc, 77900 Czech Republic; 3 Institute of Molecular and Translational Medicine, Olomouc University Hospital, Hnevotinska 1333/5, Olomouc, 77900 Czech Republic; 4 Research Group Translational Radiotheranostics, German Cancer Research Center (DKFZ), Im Neuenheimer Feld 280, 69120 Heidelberg, Germany; 5 Service Unit for Radiopharmaceuticals and Preclinical Studies, German Cancer Research Center (DKFZ), Im Neuenheimer Feld 280, 69120 Heidelberg, Germany

**Keywords:** PSMA, Prostate cancer, Preclicnical PET/CT, Theranostics, Radiopharmaceuticals

## Abstract

**Background:**

Prostate-specific membrane antigen (PSMA) is an established target for the imaging and treatment of prostate cancer. This study focused on the preclinical evaluation of three novel PSMA inhibitors—P15, P16, and P19—which were structurally modified compared to the clinically used PSMA-617. Two main strategies were pursued: a chemical approach following the so-called reversed synthetic strategy, and the replacement of the naphthyl-based linker moiety with an analogous diphenyl-based moiety. The aim was to assess the impact of these modifications on physicochemical properties, in vitro behaviour, and in vivo pharmacokinetics following radiolabelling with ⁶⁸Ga.

**Results:**

Radiolabelling of all three novel compounds with ⁶⁸Ga resulted in high radiochemical purity above 98% under physiological pH conditions and above 97% during stability testing in human plasma. All compounds exhibited hydrophilic characteristics based on partition coefficient measurements. Notable differences were observed in plasma protein binding, with P15 and P16 showing significantly lower binding compared to PSMA-617 and P19. In vitro assays using LNCaP prostate cancer cells demonstrated similar cellular uptake and internalization across all tested compounds. In vivo evaluation using Positron Emission Tomography/Computed Tomography (PET/CT) imaging in LNCaP tumour-bearing mice confirmed the tumour-targeting ability of all three inhibitors. These findings were further supported by biodistribution studies, which highlighted efficient and specific accumulation in tumour tissue. Among the tested compounds, P19 demonstrated the most promising overall profile in terms of stability, binding characteristics, and tumour uptake.

**Conclusions:**

The stereochemical modifications in the linker region significantly influenced the in vitro and in vivo behaviour of the PSMA inhibitors. Despite similar cellular uptake, differences in plasma protein binding and pharmacokinetics were evident. Among the three novel compounds, P19 emerged as a particularly promising candidate for further investigation, also indicating that the diphenyl moiety might serve as a favourable linker building block in analogy to the naphthyl moiety. Our observations suggest potential not only for diagnostic imaging with ⁶⁸Ga, but also for therapeutic applications using ^177^Lu, which offers a longer half-life suitable for delayed imaging and treatment intervals in prostate cancer management.

**Supplementary Information:**

The online version contains supplementary material available at 10.1186/s41181-025-00389-w.

## Introduction

Prostate cancer (PCa), a formidable adversary in the realm of oncology, is quietly emerging as a pervasive threat affecting millions of lives worldwide (Bray et al. 2024). Despite advances in our understanding of its biology, the complexity of PCa, especially when the metastatic disease becomes castration- and chemo-resistant after the initial treatment, requires innovative and precise therapeutic strategies. Increased prostate-specific membrane antigen (PSMA) expression on almost all types of PCa – and at the same time its limited physiological expression on majority of other tissues – makes it a very attractive molecular target for both therapeutic and imaging purposes (Al Saffar et al. 2024) In recent years, PSMA-targeted ligands have received a lot of attention, and researchers are still coming up with new ways to improve the pharmacokinetic and biodistribution properties over those currently used PSMA inhibitors (Ruigrok et al. 2021). As an outcome, a new “game changer” has entered the clinical practice in 2022, known as Pluvicto^®^ or [^177^Lu]Lu-PSMA-617. A peptidomimetic with a big impact, mainly for its use in targeted radioligand therapy with β minus-emitter lutetium-177 (Sartor et al. 2021). Despite its undeniable benefits, there are a few properties that could be improved, such as renal dose or xerostomia, which have been reported side effects in patients (Rahbar et al. 2017).

The active site of the PSMA enzyme consists of a divalent zinc and S1 and S1´ subdomains. The Glu-urea-Lys binding motif interacts primarily with the divalent zinc cation and the S1´ subdomain of PSMA, but the interaction of the linker with the lipophilic S1 subdomain (Mesters et al. 2006) is important as well. For this reason, a lot of attention among the scientific community is paid to a linker modification, which has been shown to be significant in determining pharmacokinetic and biodistribution properties in research of novel PSMA ligands (Lütje et al. 2017).

**Fig. 1:**
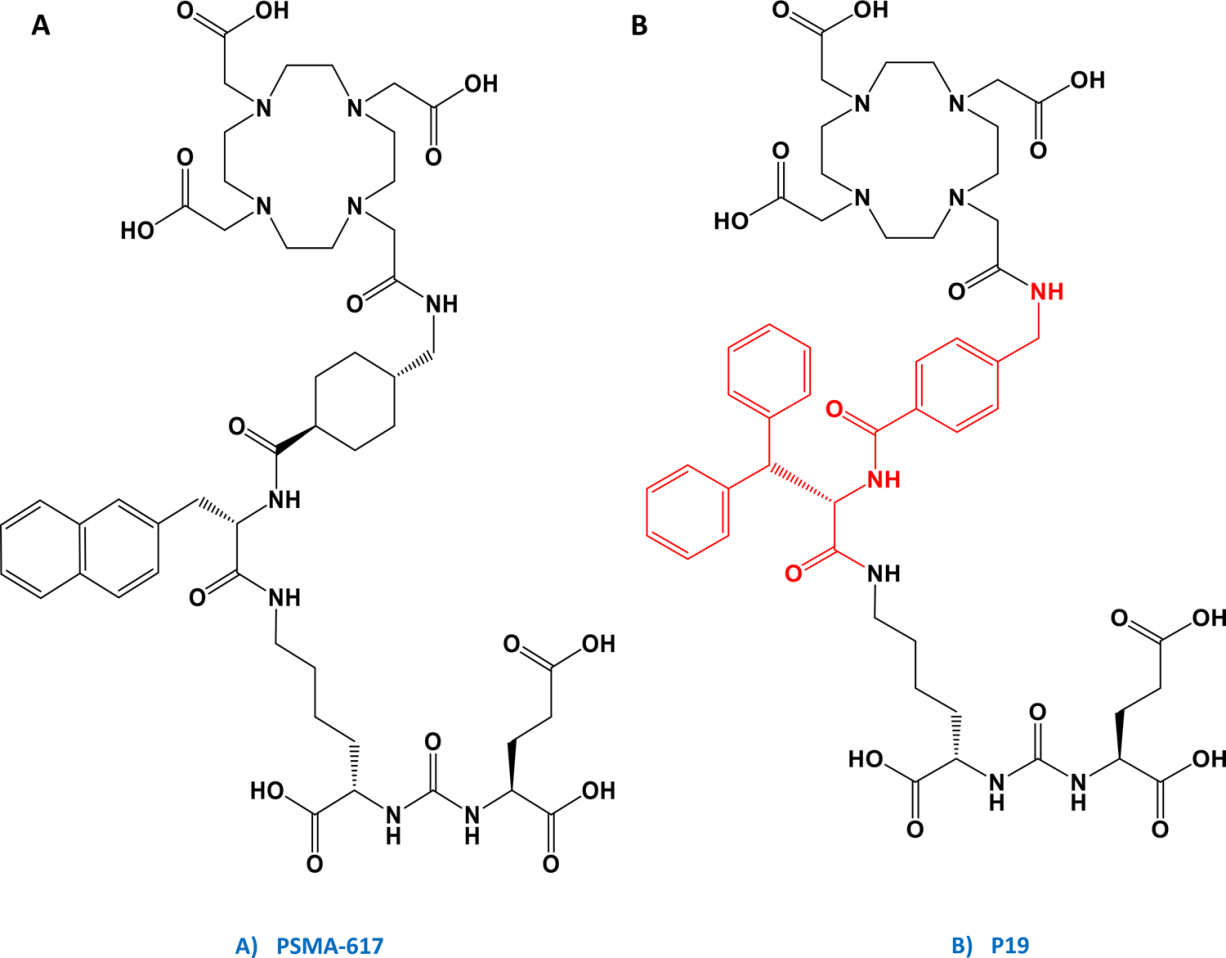
Chemical structures of synthesized Glu-ureido-Lys-based DOTA-conjugated PSMA ligands. (**A**) Gold standard compound PSMA-617 employing 2-naphthyl-L-alanine and trans-4-(aminomethyl)cyclohexyl moiety in the linker region; (**B**) Novel compound P19 employing 3,3-diphenyl-L-alanine and 4-(aminomethyl)benzoic (AMBA) moiety in the linker region

**Fig. 2:**
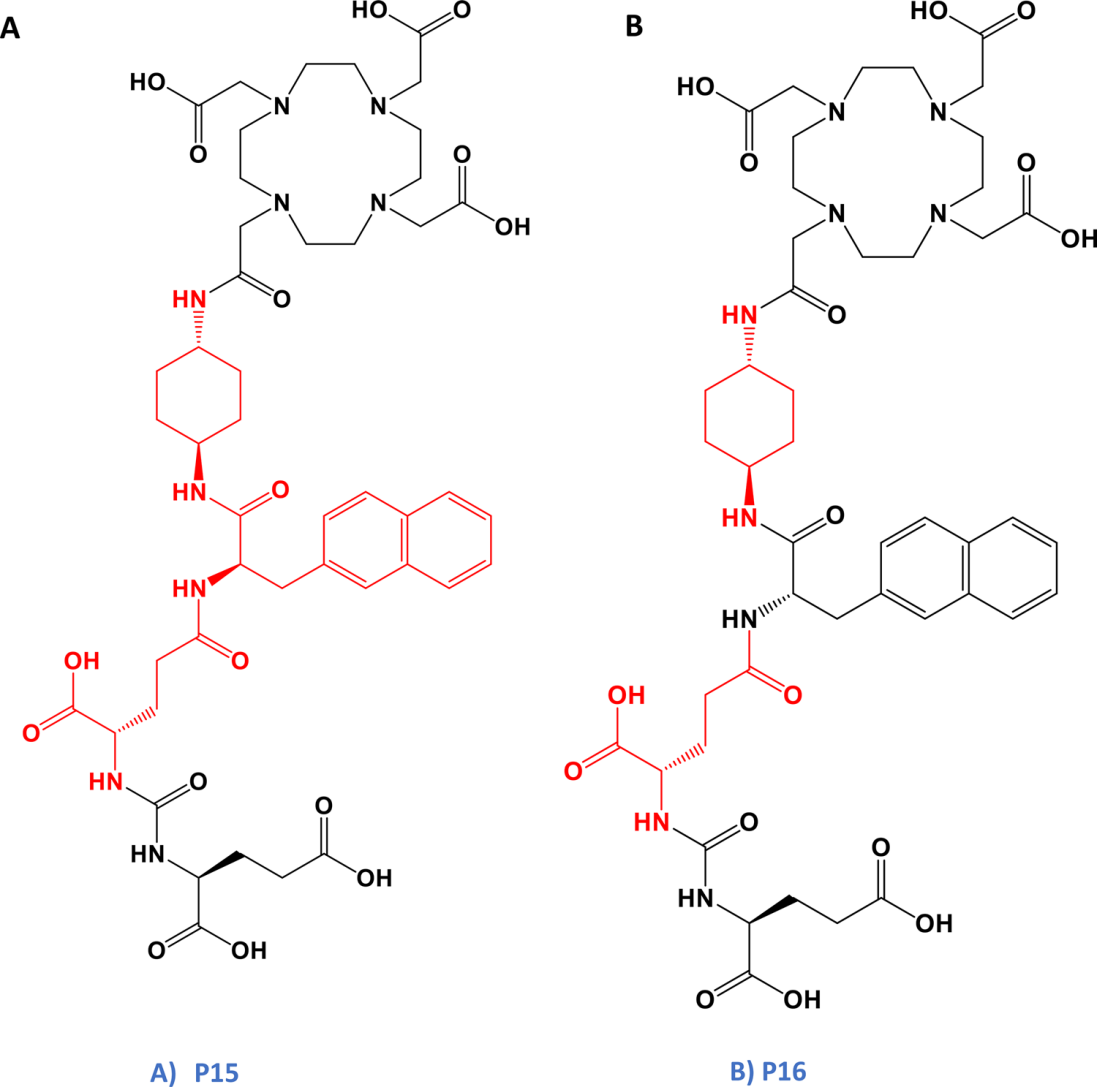
Chemical structures of synthesized Glu-ureido-Glu-based DOTA-conjugated PSMA ligands. (**A**) Novel compound P15 synthesized in a reverse direction to the standard compound PSMA-617 employing 2-naphthyl-D-Ala and trans-4-(amino) cyclohexyl moiety in the linker region (**B**) Novel compound P16 synthesized in a reverse direction to the standard compound PSMA-617 employing 2-naphthyl-L-Ala and trans-4-(aminocyclohexyl) moiety in the linker region

In this article we aimed to compare the preclinical performance of the well-established PSMA-617 with three innovative PSMA-617 analogues, distinguished by variations in the linker and/or the binding motif region. The linker moiety, connecting the pharmacophore and the radionuclide cargo, was identified as a direct contributor to binding the lipophilic S1 subdomain of the PSMA enzyme. Moreover, aromatic compounds demonstrated enhanced affinity to the S1 subdomain (Lundmark et al. 2022). Consequently, the first naphthyl-based linker moiety (2-Nal-L-Ala) was replaced for analogical diphenyl-based building block (3,3-diphenyl-L-Ala). In addition, the second cyclic linker moiety (*trans*-4-(aminomethyl)cyclohexanecarboxylic acid) was replaced with an aromatic one (4-(aminomethyl)benzoic acid), resulting in a novel analogue designated as P19 (Fig. 1). Two additional compounds, P15 and P16, were designed with distinct binding motif (Glu-urea-Glu) compared to PSMA-617 and were also synthesized using different chemical approach, following so-called reversed strategy (Fig. 2). P15 and P16 have an inverse steric orientation of the naphthylic- and cycloalkyl-based entities when comparing to the standard PSMA-617 build-up. In the newly synthesized PSMA inhibitors, we are thus mainly investigating linker modifications and steric orientation and their effect on the overall behaviour. The main aim of these modifications is to better understand structure-activity-relationship, improve imaging contrast and to reduce the potential burden on non-target organs, such as the kidneys, when employed in targeted radioligand therapy.

## Materials and methods

### Laboratory animals

Severe Combined Immunodeficiency (SCID) male mice (Envigo, Horst, The Netherlands) were used for all experiments at the age of eight weeks. All mice weighed 18–23 g and were maintained in a specific pathogen-free (SPF) animal facility with free access to water and food. Both animal housing and experiments were carried out in accordance with regulations and guidelines of the Czech Animal Protection Act (No. 246/1992), and with the approval of the Czech Ministry of Education, Youth, and Sports (MSMT-41830/2018-7 and MSMT-35035/2019-3), and the institutional Animal Welfare Committee of the Faculty of Medicine and Dentistry of Palacky University in Olomouc.

### Cell line and mice tumour model

A PSMA-positive PCa cell line was used as a model of malignant disease, namely LNCaP – CRL1740 (American Type Culture Collection - ATCC, Manassas, VA, USA). Cells were cultured in RPMI-1640 medium (Roswell Park Memorial Institute 1640, (Lonza, Switzerland)) with the addition of L-glutamine (0.3 g/L) and 20% heat-inactivated foetal bovine serum (FCSi, (Merck, USA)). At the age of eight weeks, SCID male mice were implanted subcutaneously into the right flank with 0.1 mL of approx. 10 × 10^6^ cells/mouse in a 1:1 mixture of Matrigel^®^ matrix (Corning, USA) and cell culture media. Five to six weeks after implantation, tumours with a volume of 100–300 mm^3^ were observed.

### Synthesis and QC of PSMA inhibitors

All chemicals (with purity greater than 95%) and solvents (HPLC grade) were obtained from suppliers such as abcr, Bachem, Carbolution, CheMatech, Fluka, Iris Biotech, Macrocyclics, Merck Group, Carl Roth, or Sigma Aldrich and were utilized as received without additional purification.

P15, P16, P19 and PSMA-617 (Table 1) were synthesized using 2-CT resin support (Supporting Information, Scheme S1–S4). P15, P16 and P19 were purified by semi-preparative HPLC and analyzed by ESI-MS (Supporting Information, Figure S1–S4), MALDI-MS (Supporting Information, Figure S4–S6), and analytical HPLC (Supporting Information, Figure S7–S9).

For semi-preparative and analytical HPLC, a LATEK P-402 pump coupled to a Merck Hitachi L-7420 UV/vis signal detector was employed. Column: NUCLEODUR HILIC (Macherey Nagel), 5 μm, 110 Å, 250 × 21 mm; Eluents: (A) 97% ACN + 0.2% formic acid (FA), (B) water + 0.2% FA; Solvent gradient: 0–40 min 3–50% A; Flow: 15 mL/min; Wavelength: 214 nm; Temperature: RT.

The mass spectra of PSMA-617 and all three novel compounds were recorded using an Esquire 600 (Bruker) mass spectrometer with an ion trap, as well as an LT2 Plus (Scientific Analysis Instruments, SAI) mass spectrometer equipped with a time-of-flight (TOF) detector.

**Table 1:** Analytical data of novel PSMA ligands P15, P16 and P19 as well as standard PSMA-617

	Structure	Molecular weight [g/mol]	Yield [mg/%]	Purity [%]
P15	Glu-urea-Glu-2-Nal-D-Ala-NH-CHX-NH-DOTA	1000.7	215/(59)^*^ 12/(23)	> 99%
P16	Glu-urea-Glu-2-Nal-L-Ala-NH-CHX-NH-DOTA	1000.7	220/(15)^*^ 14/(20)	> 99%
P19	Glu-urea-Lys-3,3-diphenyl-L-Ala-AMBA-DOTA	1062.5	125/(38)	> 99%
PSMA-617	Glu-urea-Lys-2-Nal-L-Ala-CHX-DOTA	1042.1	140/(33)	> 95%

^*^Yield corresponds to the synthesis without DOTA chelator. For the final conjugation step, only part of the precursor was used

### Radiolabelling of PSMA inhibitors

Ga-68 labelling was performed using ^68^GaCl_3_ eluted from a ^68^Ge/^68^Ga generator (type IGG100, Eckert & Ziegler, Berlin, Germany) with 0.1 M HCl (Fluka, Buchs, Switzerland). Typically, 5 µg PSMA inhibitor (1 µg/µL in H_2_O) was mixed with 30 µL sodium acetate (155 mg/mL in water, 1.14 M). Subsequently, 300 µL ^68^Ga eluate (15–40 MBq) was added. The resulting mixture (pH 3–4) was incubated in a dry bath for 5 min at 95 °C. After incubation 100 µL of sodium acetate was added to increase the pH to 6–7.

The radiolabelling conditions, time of incubation and amount of PSMA inhibitor were estimated experimentally. From the incubation time-points of 1, 5 and 10 min, the 5 min time-point was chosen as optimal because it was the shortest to have the radiochemical yield greater than 99% (Supporting Information, Table S1). The 5 µg of PSMA inhibitor were chosen considering the radiochemical yield greater than 99% with molar activity 8 GBq/µmol (Supporting Information, Table S2).

### HPLC analysis of radiochemical purity

Quality control, i.e. radiochemical purity, of the ^68^Ga-labelled PSMA inhibitors was evaluated by analytical high performance liquid chromatography (HPLC; Dionex UltiMate 3000, Thermo Scientific, Waltham, Massachusetts, USA) using the following equipment: ACE 3 C18 column; 150 × 3 mm, 3 μm (Advanced Chromatography Technologies Ltd, Aberdeen, UK), with gradient elution A: H_2_O/acetonitrile (ACN); B:0.1% trifluoroacetic acid (TFA)/H_2_O: 0–3 min, 0% ACN; 3–10 min, 0 − 50% ACN; 10–13 min, 50 − 80% ACN; 13–15 min, 80–0% ACN, for 15 min at a flow rate of 1 mL/min. Radiometric detection was performed using a GABI Star radiodetector (Raytest GmbH, Straubenhardt, Germany) and for chromatogram quantification Chromatography Data System (CDS) - Chromeleon software (Thermo Scientific, USA) was used. The actual sample injection was performed using a Hamilton microinjector (Hamilton, USA).

### In vitro assays

Radiochemical stability in a physiological pH water environment was determined as follows: 100 µL of acetate buffer (1.1 mol/L) was added to the labelled preparation and the pH was then checked with a pH indicator paper to be 7. The mixture was further incubated at 37 °C and after 30, 60 and 120 min, 10 µL aliquot was used for HPLC analysis of radiochemical purity.

Plasma stability was determined as follows. To 100 µL of ⁶⁸Ga-P15 with radioactivity of 45 kBq/100 µL, 400 µL of human plasma was added and mixed. The mixture was incubated at 37 °C and 100 µL samples were taken at 30, 60 and 120 min. These samples were then mixed with 200 µL of acetonitrile and placed on a shaker for 1 min. The sample was then centrifuged at 15,000 rpm for 3 min. After centrifugation, the supernatant was collected for radiochemical purity analysis by HPLC. The same procedure was applied when determining plasma stability of ⁶⁸Ga-P16, ⁶⁸Ga-P19, and ⁶⁸Ga-PSMA-617.

Binding to human plasma proteins was determined following this procedure: 10 µL of the ⁶⁸Ga-P15 with radioactivity of 45 kBq/100 µL was mixed with 190 µL of human plasma. The sample was then incubated at 37 °C. After 0, 30, 60 and 120 min 25 µL of the sample was collected, applied to the appropriate column and centrifuged at 2000 g for 2 minutes. The radioactivity of the samples was then measured using a gamma counter 2480 Wizard^2^ (PerkinElmer, Waltham, MA, USA), and then the binding to plasma proteins was calculated using the following formula % plasma protein binding = (A_elution_/A_total_)×100. The same procedure was applied when determining binding to plasma proteins of ⁶⁸Ga-P16, ⁶⁸Ga-P19, and ⁶⁸Ga-PSMA-617.

Octanol/water partition coefficient (P) was determined as described before (Petrik et al. 2012). Briefly, 600 µL PBS was added to the total volume of the ⁶⁸Ga-P15. New tube was filled with 50 µL of the above-mentioned solution, 500 µL octanol and 450 µL PBS. Five more samples were prepared in this way and then placed on an orbital shaker for 20 min. The samples were then centrifuged at 15,000 rpm for 1 min. After centrifugation, two samples were taken from each tube, one from the octanol and the other from the aqueous phase. The radioactivity of these samples was measured using an automated gamma computer and the formula log P = log(A_octanol_/A_H2O_) was employed to calculate log P. The same procedure was applied when determining the partition coefficient of ⁶⁸Ga-P16, ⁶⁸Ga-P19, and ⁶⁸Ga-PSMA-617.

The competition assay, LNCaP cells were seeded into poly-L-lysine coated 24-well plates in amount of 0.5 × 106 cells/well 24 h prior to the competition assay. The tested ligands (^68^Ga-P15, ^68^Ga-P16 and ^68^Ga-P19) were added to the wells in concentrations 0, 1, 5, 10, 25, 50, 100, 500, 1000 and 5000 nM in triplicates. Afterwards, ^68^Ga-labelled PSMA-617 was added to each well so its final concentration was 5 nM. All the ligands in this assay were dissolved in the buffer containing 25 mM Tris-HCl, 5.4 mM KCl, 1.8 mM CaCl2, 0.8 mM MgSO4, 5 mM glucose, and 140 mM NaCl in H2O (pH adjusted to 7.4). ^68^Ga-PSMA-617 was incubated on cells for 60 min at 37 °C followed by its removal and washing the cells with PBS. Finally, the cells were disintegrated using 0.1 M NaOH and transferred into the scintillation vials for the gamma counting. The IC50 values were calculated by fitting the data using a nonlinear regression algorithm (GraphPad PRISM 7.0) and the data were assessed by t-test.

### In vivo studies

The eight-week-old male SCID mice (Charles River, Massachusetts, USA) were used to generate the mouse tumour model. The implantation of 10 million LNCaP cells (ATCC, Virginia, USA) per mouse in a 1:1 mixture of Matrigel matrix (Corning, New York, USA) was performed subcutaneously behind the right shoulder of the mouse. Tumours of adequate size, representing a volume of 100–300 mm³, were observed after five to six weeks.

Ex vivo biodistribution of PSMA inhibitors in tumor-bearing mice was conducted as follows. Concisely, 30 µL of acetate buffer was added to the each ⁶⁸Ga -labelled inhibitor, namely: ⁶⁸Ga-P15, ⁶⁸Ga-P16, ⁶⁸Ga-P19, and ⁶⁸Ga-PSMA-617 to achieve physiological pH. Mice were r.o. injected with 100 µL of labelled inhibitor (2 MBq, approximately 0.5 µg of the inhibitor). Animals were divided into two groups for biodistribution studies at 1 and 2 h after administration of the respective ^68^Ga-labelled PSMA inhibitor. The animals were sacrificed and autopsied after euthanasia, and organs: spleen, pancreas, stomach, intestine, kidney, liver, heart, lung, muscle, bone, and tumour were collected in scintillation vials. The vials containing the samples were weighed and measured in a Wizard2 gamma counter (PerkinElmer, USA). The measured values were then adjusted for radionuclide decay and expressed as a percentage of administered dose per gram of organ (%ID/g). The NanoScan^®^ hybrid Positron Emission Tomography/Computed Tomography (PET/CT) machine (Mediso, Budapest, Hungary) was used for in vivo imaging specifically whole-body helical PET/CT scans. Imaging settings were: single field of view (FOV) PET scans (98.5 mm) which took 10 min for each mouse and helical CT scans (50 kVp/980 µA, 720 projections) both took place one hour after the administration. Each mouse received an injection of approximately 10 MBq of tested radiopharmaceutical. To prevent the mice’s eyes from drying out during anaesthesia eye ointment (Ophthalmo-Azulen, Zentiva, CR) was applied. Image reconstruction was performed using Mediso Tera-Tomo 3D PET iterative reconstruction (Mediso Medical Imaging Systems, Budapest, Hungary). Both reconstruction and data evaluation were performed in Mediso InterView FUSION software (Mediso Medical Imaging Systems, Budapest, Hungary) (Mediso, Budapest, Hungary). Final images were normalised to injected activity and animal weight. All organs with radiopharmaceutical accumulation were roughly delineated on the images, while their SUVmax value was recorded. The SUV_41%_ (Standardized Uptake Value 41%) was then calculated as 41% of each SUVmax value of given hotspot and the ROI was delineated based on this value (Boellaard et al. 2015). All images were exported in maximum intensity projection (MIP) mode. The results from the ex vivo biodistribution study were subjected to statistical analysis, namely unpaired Student’s *t*-test (Microsoft Excel 365, USA).

## Results

### Synthesis

The 10-steps synthesis of P15, P16, P19 and PSMA-617 on a solid phase was successfully performed. All four compounds were obtained with yields ranging from 20% for reversely synthesises compounds and from 33% for classical synthesis. All compounds were gained at high purity (> 99% for novel compounds and > 95% for reference compound PSMA-617) (Table 1). The identity of all compounds was confirmed by ESI-MS, MALDI-MS and HPLC (Figures S1-S9, Supplemental Information).

### Labelling conditions

Firstly, the labelling conditions were evaluated whether the 15 min at 95 °C as Benešová et al. evaluated for PSMA-617 (Benešová et al. 2015) could be modified for the new analogues regarding the ⁶⁸Ga half-life which only is 68 min. From the examined time-points of 1, 5 and 10 min, the 5 min time-point was chosen as optimal because it was the shortest to have the radiochemical yield greater than 99% (Supporting Information, Table S1). An amount of PSMA inhibitor for labelling with ⁶⁸Ga was evaluated experimentally (Supporting Information, Table S2), where we established 5 µg had the radiochemical yield greater than 99% with molar activity 8 GBq/µmol (Supporting Information, Figures S10-S13).

### Assessment of in vitro parameters

#### Determination of stability in physiological pH environment

For the determination of radiochemical stability, inhibitors were labelled with ^68^Ga and incubated at pH 7 in solution of sodium acetate (1.14 mol/l) as well as in human blood serum at 37 °C for 30, 60 and 120 min. All inhibitors had radiochemical purity greater than 97% in physiological pH and in human blood serum (Table 2).

#### Determination of plasma protein binding

Determination of ^68^Ga-labelled PSMA inhibitors binding to plasma proteins is shown in Table 2, where inhibitor [^68^Ga]Ga-P19 shows the highest plasma protein binding rate, 59% after 30 min of the incubation and 58% thereafter. Inhibitor [^68^Ga]Ga-P16 shows the lowest plasma protein binding rates of all inhibitors tested, ranging from 10% at baseline to 13% after two hours incubation. [^68^Ga]Ga-P15 shows the greatest change in plasma protein binding over time, from 29% after 1 h to 40% after 2 h. After two hours of incubation, the gold standard, PSMA-617 showed only a negligible increase from 53% to 54% in plasma protein binding.

#### Determination of partition coefficient (P)

All studied inhibitors showed hydrophilic properties with partition coefficients (log P) less than − 3: ranging from − 3.07 for P16; − 3.41 for P19; − 3.46 for PSMA-617 and − 3.71 for P15 (Table 2). Which suggests that structure modifications that were made do not compromise hydrophilic nature of compounds.

### Competition assay

IC50 values for [^68^Ga]Ga-P15, [^68^Ga]Ga-P16 and [^68^Ga]Ga-P19 were determined as follows 97.3, 4.0 and 95.6 nmol/l in the competition with [⁶⁸Ga]Ga-PSMA-617.

**Table 2:** In vitro characterization of [^68^Ga]Ga-PSMA-617, -P15, -P16 and -P19. Log P, protein binding stability in human serum and PBS at physiological pH

[^68^Ga]Ga-PSMA inhibitor	Log P (mean ± SD *n* = 6)	Incubation time (min)	Protein binding (%) (*n* = 2)	Stability in human serum (%) (*n* = 1)	Stability in Physiological pH (%) (*n* = 1)
[^68^Ga]Ga-PSMA-617	–-3,46 ± 0,04	0	52.6%	98.4%	98.4%
		30	52.9%	97.8%	98.5%
		60	53.3%	98.2%	98.9%
		120	54.3%	98.7%	98.5%
[^68^Ga]Ga-P15	–3,71 ± 0,14	0	26.7%	99.0%	99.0%
		30	27.0%	98.9%	98.8%
		60	29.4%	99.8%	98.7%
		120	39.9%	99.4%	99.2%
[^68^Ga]Ga-P16	–3,07 ± 0,87	0	10.2%	99.0%	99.3%
		30	10.9%	98.8%	99.1%
		60	10.8%	98.3%	99.3%
		120	13.4%	97.3%	99.3%
[^68^Ga]Ga-P19	–3,41 ± 0,13	0	56.6%	99.0%	99.8%
		30	58.8%	99.3%	99.5%
		60	57.7%	99.8%	99.5%
		120	58.2%	99.9%	99.6%

Values for protein binding are expressed as percentage of protein-bound activity of the total activity used

### Ex vivo biodistribution studies

**Fig. 3:**
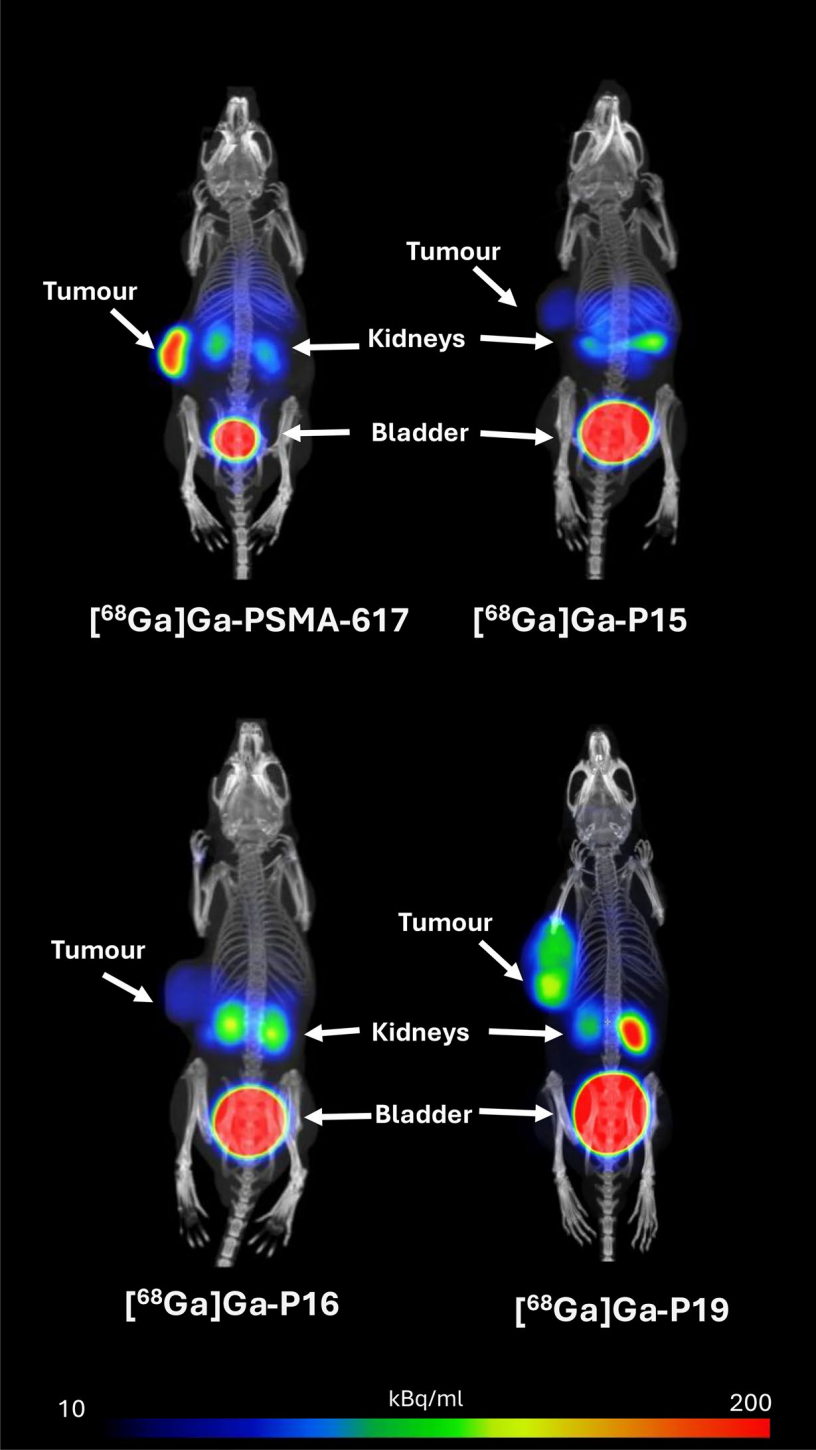
PET/CT image of LNCaP tumour-bearing mice injected with 10 MBq [^68^Ga]Ga-PSMA-617, -P15, -P16 and -P19 inhibitors. All images were acquired in MIP mode,1 h p.i

Ex vivo biodistribution studies were performed in mouse xenograft model bearing LNCaP tumour. In the Fig. 3, we show representative PET/CT images of mice taken in MIP mode, each taken one-hour post-injection (p.i.) of the respective ^68^Ga-labelled PSMA inhibitor. The hydrophilicity of the PSMA inhibitors was also confirmed by PET/CT images, as the highest activity on the images was always seen in the bladder, thus a rapid excretion of ^68^Ga-labelled PSMA inhibitors via urine was observed (Fig. 3). Accumulation of the ^68^Ga-labelled PSMA inhibitors in other organs was negligible. When ^68^Ga-labelled PSMA inhibitors were used, the tumour could always be distinguished from the surrounding tissue.

**Fig. 4:**
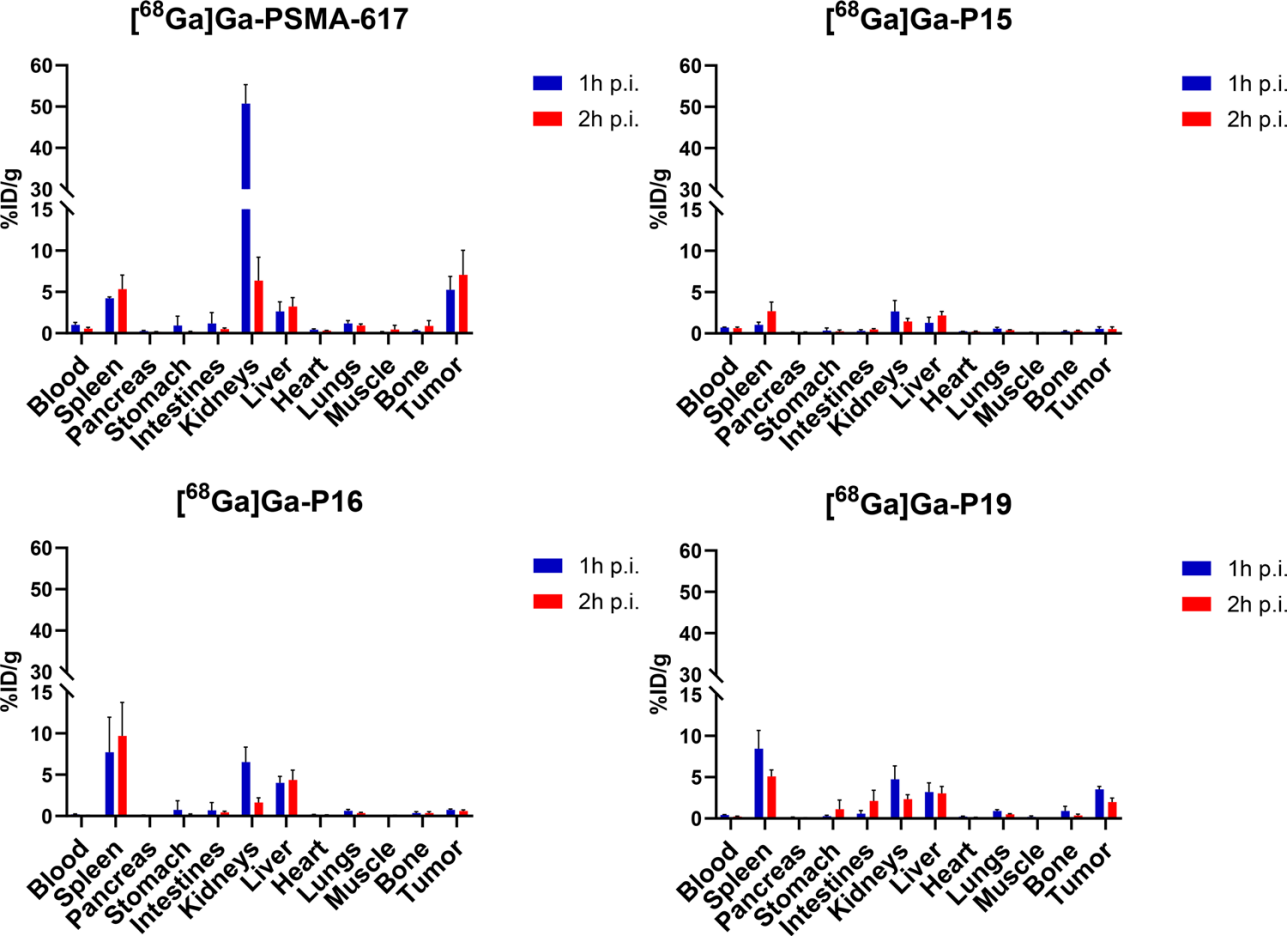
Biodistribution of [^68^Ga]Ga-PSMA-617, -P15, -P16 and -P19 inhibitors in LNCaP tumor-bearing mice. Values are shown as the mean of the percentage of administered dose per gram of the organ (%ID/g) ± standard deviation (SD). For the respective organ, the first column always represents the values at one hour after administration and the second column represents the values at two hours after administration (*n* = 4)

[^68^Ga]Ga-PSMA-617 demonstrated the highest uptake in the tumour (Fig. 4); however, notably, the highest values of the administered dose per gram were also measured in the kidneys, particularly one-hour p.i. (Fig. 4). On the other hand, the [^68^Ga]Ga-P15 inhibitor exhibited the lowest accumulation in the tumour, with only 0.5% detected two-hours p.i. (Fig. 4).

For the [^68^Ga]Ga-P16 inhibitor, the highest accumulation of all organs was observed in the spleen with 7.7% ID/g and 9.6% ID/g at one- and two-hours p.i., respectively (Fig. 4). Accumulation in the tumour for [^68^Ga]Ga-P19 biodistribution was more than half that of PSMA-617 at one-hour p.i. (3.5% ID/g vs. 5.3% ID/g). However, this decreased to 2.0% ID/g at two-hours p.i.

**Fig. 5:**
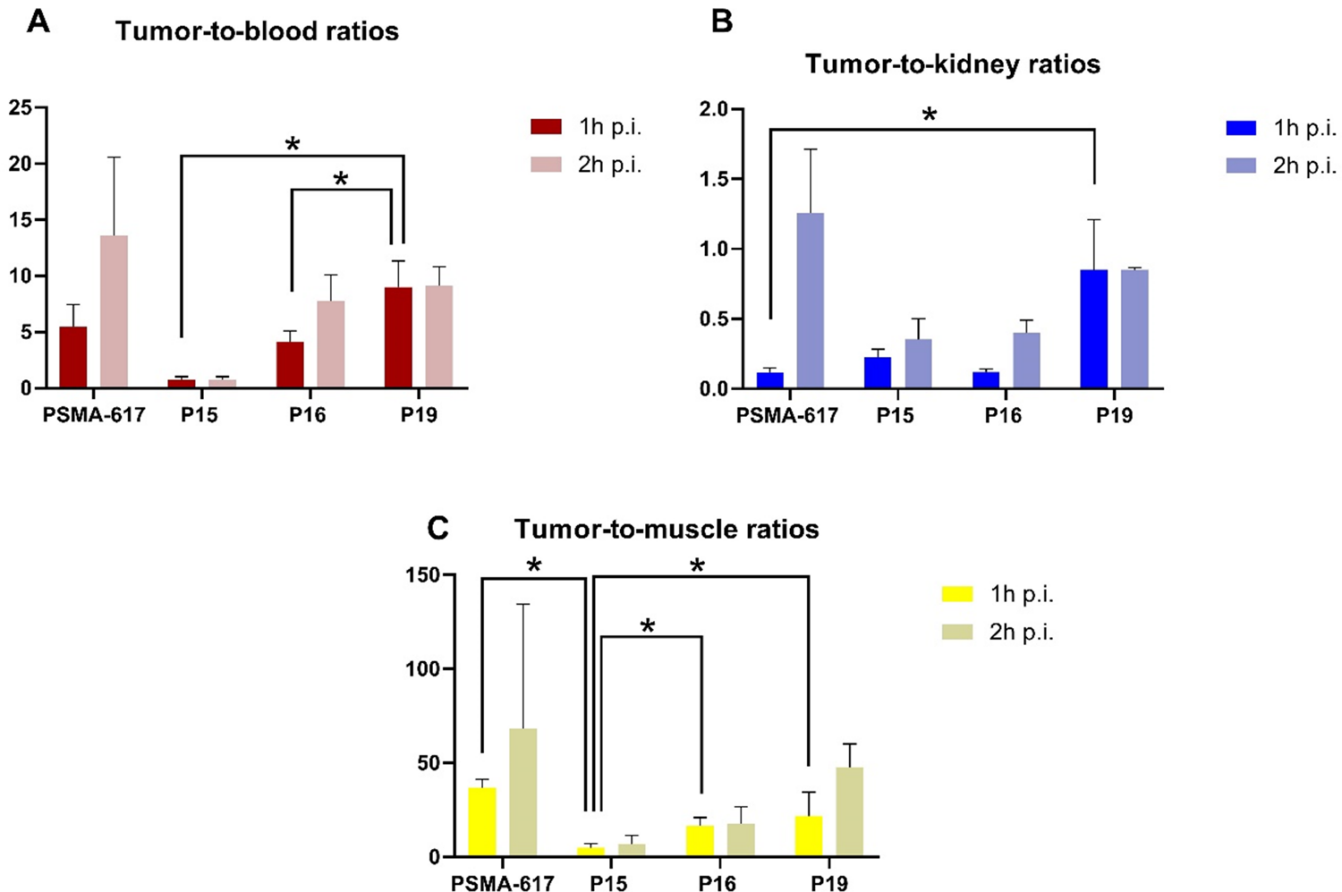
Accumulation ratio of [^68^Ga]Ga-PSMA-617, -P15, -P16 and -P19 inhibitors in **A**) tumour-to-blood, **B**) tumour-to-kidney and **C**) tumour-to-muscle at 1 h and 2 h p.i. Values are shown as the mean ± standard deviations (*n* = 4), (* *p* < 0.05)

The different ratios of the percentage of the administered dose per gram of ^68^Ga-labelled PSMA inhibitors in the tumour versus the three different organs blood, muscle and kidneys can be seen in Fig. 5. These data were obtained from the ex vivo biodistribution data (Table S3, Supporting Information). The tumour-to-blood ratio is higher for [^68^Ga]Ga-P19 when compared to [^68^Ga]Ga-P15 and [^68^Ga]Ga-P16 inhibitors in both cases. Likewise, when compared to the standard at one-hour p.i., [^68^Ga]Ga-P19 and [^68^Ga]Ga-PSMA-617 had values of 9.0 ± 2.3 and 5.5 ± 2.0, respectively (Fig. 5). [^68^Ga]Ga-P19 also showed more favourable renal accumulation values at one-hour p.i. 0.85 ± 0.36 vs. 0.12 ± 0.03 (tumour-to-kidney), thus it can be less harmful to the kidneys at the shorter time points than the [^68^Ga]Ga-PSMA-617, which has better tumour-to-kidney ratio at two hours post injection 0.85 ± 0.02 vs. 1.26 ± 0.46, respectively. However, at longer time point, at two-hours p.i., [^68^Ga]Ga-PSMA-617 had better both tumour-to-background ratios when compared with inhibitor P19; 13.6 ± 7.0 vs. 9.1 ± 1.7 (tumour-to-blood) and 68.4 ± 66.1 vs. 47.8 ± 12.4 (tumour-to-muscle), but the difference is not statistically significant.

### In vivo imaging

The small animal single field of view (FOV) PET/CT scan at one-hour p.i. (Fig. 3) revealed ability of all tested inhibitors to accumulate in LNCaP tumours, therefore visualize them. As suggested the tumour-to-muscle ratio, among the new tested PSMA inhibitors, [^68^Ga]Ga-P19 showed the most favourable contrast on the PET/CT scan, although it didn´t surpassed the gold standard, [^68^Ga]Ga-PSMA-617 still showed superior contrast abilities.

## Discussion

The increased expression of PSMA on almost all types of PCa and its simultaneous limited physiological expression on majority of other tissues makes it a very attractive molecular target for both therapeutic and imaging purposes (Ross et al. 2003). In recent years, PSMA inhibitors have attracted considerable attention, with ongoing research focused on developing novel strategies to enhance their pharmacokinetic properties compared to those of existing inhibitors, whether for imaging, such as PSMA-11, DCFPyL, MIP-1404, or for theranostic use, such as MIP-1095, PSMA-617 and PSMA I&T (Ruigrok et al. 2021). PSMA enzyme active site consists of a divalent zinc and S1 and S1' subdomain. The Glu-urea-Lys binding motif primarily interacts with the divalent zinc cation and the S1' subdomain of PSMA. However, the interaction of the linker itself with the lipophilic S1 subdomain appears to be equally important (Benešová et al. 2016). Therefore, linker modification currently receives much attention in the scientific community and has been shown to be important in determining pharmacokinetic properties in novel PSMA ligand research (Lütje et al. 2017). We have synthesized novel PSMA inhibitors that all share the DOTA chelator and the Glu-ureido-based binding motif. Specifically, [^68^Ga]Ga-P15 and [^68^Ga]Ga-P16 employs a Glu-urea-Glu binding motif and [^68^Ga]Ga-PSMA-617 and [^68^Ga]Ga-P19 possess Glu-urea-Lys binding motif (Figs. 1 and 2). The linker modifications, mainly in the terms of their influence on the overall performance of the molecule, are primarily investigated in the newly synthesised PSMA ligands. Improving PET/CT contrast and reduction of potential burden on non-target organs is the main goal of these modifications.

The labelling time and amount of PSMA inhibitor to reliably achieve a radiochemical purity of ^68^Ga-labelled PSMA inhibitors of at least 95% needed to be determined. Labelling was consistently performed at 95 °C, as this temperature represents a standard condition for radiometal complexation into the DOTA chelator(Benešová et al. 2015; Price and Orvig 2014). Our goal was to achieve radiochemical purity greater than 99%, which we were able to achieve in both 5 min and 10 min labelling (Table S1). However, given the short half-life of ^68^Ga, we selected a 5 min labelling time. The optimal amount of PSMA inhibitor for labelling was 5 µg, which allowed to achieve radiochemical purity above 99% (Table S2), so we chose this amount for all further labellings with molar activity 8 GBq/µmol.

Regarding the stability of the investigated PSMA inhibitors in a physiological pH water environment, P19 achieved the highest stability of all PSMA inhibitors tested, including the standard PSMA-617, as shown in Table 2. However, all inhibitors were highly stable in a physiological pH environment, as their radiochemical purity was greater than 98% at each time-point tested (Table 2). Our results are also in line with the work of Benesova et al., where the [^68^Ga]Ga-PSMA-617 showed the same stability in a physiological pH environment as in the study performed by us (Benešová et al. 2015). As [^68^Ga]Ga-P15 and [^68^Ga]Ga-P16 are synthesised in the opposite direction compared to [^68^Ga]Ga-PSMA-617, differing only in one amino acid and stereochemistry, we expected comparable stability, which was confirmed (Table 2).

Stability studies in human blood plasma have shown that all the PSMA inhibitors are stable in this environment, with radiochemical purity values above 97%. Inhibitor [^68^Ga]Ga-P16, which also always had radiochemical purity values above 97% (Table 2), was the only inhibitor to show a decreasing trend over time. The binding of radiotracers to plasma proteins is considered crucial to improve their pharmacokinetic profile, i.e. their distribution in target and non-target tissues. Drugs with enhanced binding capacity to plasma proteins circulate longer in the blood and could show higher tumour-to-background levels (Müller et al. 2013).

The binding rate to human plasma proteins is approximately 54% for [^68^Ga]Ga-PSMA-617, according to our results, a binding to plasma proteins of 59% is reported in the available literature (Benešová et al. 2018). Such binding is generally not considered to be high and PSMA-617 does not contain a component in its structure that explicitly targets the plasma proteins, although it was described that naphthylamine-similar components bind near Trp-134 into albumin’s subdomain IA (Ojha and Das 2010). Therefore, the biodistribution mode is influenced by hydrophilicity rather than passive diffusion, and structural modifications targeting plasma proteins could prolong its half-life. From bloodstream, they are taken up by cells expressing the PSMA enzyme on their surface and subsequently internalised by clathrin-mediated endocytosis, leading to their accumulation in the cytoplasm of the cells (Kratochwil et al. 2016) Inhibitor [^68^Ga]Ga-P19 showed similar levels of binding to human plasma proteins as [^68^Ga]Ga-PSMA-617. Neither inhibitor [^68^Ga]Ga-P15 nor [^68^Ga]Ga-P16 reached levels above 40%, with the latter reaching plasma protein binding levels less then binding of [^68^Ga]Ga-P15, but both increased over time (Table 2). We conclude that conformational changes (Naphtyl-D-alanine vs. Naphtyl-L-alanine) influence plasma protein binding, as this is the only difference between inhibitors [^68^Ga]Ga-P15 and [^68^Ga]Ga-P16 (Table 2). However, it must be noted that the binding of [^68^Ga]Ga-PSMA-617 to human plasma proteins is much higher compared to its binding to mouse serum proteins (59% vs. 6%) which may have relevant implications for biodistribution in the mouse model, such as lower tumour accumulation, but of course also lower activity in the blood compared to the human situation (Umbricht et al. 2018).

Another parameter investigated was the octanol/water partition coefficient, which quantifies the ability of a compound to disperse between a non-polar solvent, such as octanol, and water, which is a polar solvent. This parameter is often used as a measure of the lipophilicity or hydrophobicity of a compound. The hydrophobicity of a compound is an important characteristic that influence its pharmacokinetic properties, including its absorption, distribution, metabolism, and elimination from the body (Lipinski 2000). PSMA radioligands are generally considered to be hydrophilic molecules, resulting in rapid distribution within the body with excretion preferentially via the renal route (Umbricht et al. 2018). All investigated PSMA inhibitors were evaluated as hydrophilic based on our results, with negative octanol/water partition coefficient values, all below − 3 (Table 2). These values are in agreement with data from the available literature, where PSMA-617 was evaluated as a hydrophilic molecule (Umbricht et al. 2018). The hydrophilic PET tracers generally have faster clearance from well-perfused organs compared to the lipophilic ones, leading to higher tumour-to-background values (Zhu et al. 2022). The low tumour/background ratio value in this case is mainly attributed to the stereochemistry in the linker region, as this has also been shown to be critical for tumour uptake of the inhibitor (Benešová et al. 2022). In the paper by Benesova et al. it was shown that conformational changes between the L- and D-form, in this case in the 2-naphthyl-D-alanine and 2-naphthyl-L-alanine region, significantly altered the pharmacokinetic properties of the PSMA inhibitor, in particular the D-form had a significantly reduced capacity for both inhibitory potential and specific internalisation (Benešová et al. 2016).

Determined IC50 values indicates the best in vitro affinity for [^68^Ga]Ga-P16 with its IC50 in nanomolar range which was significantly lower compared to the other two IC50 values. Despite the fact that [^68^Ga]Ga-P15 is chemically identical to [^68^Ga]Ga-P16 with the exception of stereochemistry of the alanine the IC50 of [^68^Ga]Ga-P15 is almost 25-fold higher than that of [^68^Ga]Ga-P16. This shows how stereochemistry plays crucial role in the means of binding ability. Nevertheless, established in vitro affinities are not in accordance with in vivo measured tumor accumulation, where we can see the best tumor uptake for [^68^Ga]Ga-P19. This finding can be subject of further exploration thus there are several factors what can explain such discrepancy e.g. difference in vitro vs. in vivo stability or different internalization rate.

We note that all investigated ^68^Ga-labelled PSMA inhibitors were effective in imaging LNCaP tumours by PET/CT as early as 1 h p.i., as shown by representative images of tumour-bearing mice (Fig. 3). In all cases, we were able to distinguish the tumour from the surrounding tissue based on the tracer accumulation in the tumour. We observed the worst tumour contrast on PET/CT images in the case of inhibitor [^68^Ga]Ga-P15, which was subsequently confirmed by the biodistribution study and the resulting tumour-to-background ratios, whether blood or muscle. The tumour-to-muscle ratio for inhibitor [^68^Ga]Ga-P15 was the lowest of all PSMA inhibitors tested, more than 7-fold lower than the gold standard, [^68^Ga]Ga-PSMA-617 (Fig. 5). [^68^Ga]Ga-P15 tumour uptake was also the lowest of all inhibitors tested, being more than 5-fold lower than its L-isomer, inhibitor P16, even at one-hour p.i., and up to 10-fold lower than [^68^Ga]Ga-P16 at two-hours p.i. (Fig. 4). Comparing the inhibitor P15 with the gold standard [^68^Ga]Ga-PSMA-617, the specific accumulation was 7-fold lower one-hour p.i. and up to more than 17.5-fold lower two-hours p.i. of the inhibitor (Fig. 4).

These results are consistent with previous findings on the significant impact of stereochemistry on the degree of specificity of PSMA inhibitor binding to tumours (Benešová et al. 2016). The cause may be that P15 and P16 have a Glu-urea-Glu binding motif, which generally has a lower PSMA affinity (Glu-urea-Glu K_i_ = 47 nM (Kozikowski et al. 2001), Glu-urea-Lys K_i_ = 15.25 nM (Banerjee et al. 2008), PSMA-617 K_i_ = 2.34 nM (Benešová et al. 2015), i.e. the ability to bind to the active site of PSMA, than Glu-urea-Lys, which has both PSMA-617 and P19 (Kozikowski et al. 2001). However, in the case of [^68^Ga]Ga-PSMA-617 and [^68^Ga]Ga-P19, we were able to distinguish the tumour from the surrounding tissue much better on PET/CT images (Fig. 3) compared to [^68^Ga]Ga-P15 and [^68^Ga]Ga-P16. In addition to their better accumulation in the tumour confirmed in the PET/CT images (Fig. 3) the same was recorded by the biodistribution study (Fig. 4) and the resulting data of tumour-to-blood, muscle and kidney ratios (Fig. 5).

The tumour-to-the blood ratio one hour after administration was more than 1.5 times higher for [^68^Ga]Ga-P19 than for [^68^Ga]Ga-PSMA-617. Two hours after administration, however, the situation had changed and PSMA-617 had an almost 1.5-fold higher tumour-to-background ratio than [^68^Ga]Ga-P19. The better accumulation of the [^68^Ga]Ga-P19 inhibitor in the tumour at one hour after administration can be mainly attributed to the AMBA (4-aminomethylbenzoic acid) moiety in its structure, which adds a third aromatic ring — the optimal number for tumour uptake (Benešová et al. 2016). This theory is also supported by the study by Liu et al., where the authors suggest that the binding site of the PSMA enzyme interacts with hydrophobic aromatic rings in the inhibitor molecule (Liu et al. 2008). However, the number of atoms between the urea moiety of the PSMA inhibitor and the aromatic moiety of the molecule is also important. If there are seven atoms between them, the aromatic part of the inhibitor will fit well into the S1 hydrophobic part of the PSMA enzyme (Nakajima et al. 2018).

The better contrast of [^68^Ga]Ga-P19 when compared to [^68^Ga]Ga-P15 or [^68^Ga]Ga-P16 in the images was also confirmed by the data from the biodistribution study, namely the tumour-to-muscle ratio (Fig. 5). In this comparison, [^68^Ga]Ga-PSMA-617 has more than twice the values of inhibitor [^68^Ga]Ga-P16, more than 1.6 times the levels of inhibitor [^68^Ga]Ga-P19 and almost 7.5 times the levels of inhibitor [^68^Ga]Ga-P15 one-hour p.i. Inhibitor [^68^Ga]Ga-P19 has a higher tumour-to-muscle ratio at two-hours p.i. at one-hour p.i., and the values are closer to those of [^68^Ga]Ga-PSMA-617. We attribute this mainly to the reasons mentioned above, and therefore mainly to the presence of the AMBA moiety in the structure of P19, and to the rapid pharmacokinetics of the PSMA inhibitors. The excretion of PSMA inhibitors is mainly renal due to their hydrophilicity. In the renal environment, they are subject to glomerular filtration, where molecular weight is an important factor. Molecules below 20 kDa are usually not reabsorbed and are therefore excreted in the urine (van der Gaag et al. 2022).

This was confirmed by PET/CT images, which consistently showed the bladder as the organ with the highest activity (Fig. 3). It cannot be overlooked that at the same time as the accumulation of [^68^Ga]Ga-P19 inhibitor increased in the tumour, its level also increased in the spleen, as can be seen in Fig. 4. However, its level in the kidney one hour after administration, which is one of the most critical organs in PSMA inhibitor endoradiotherapy, was up to 7-times lower compared to [^68^Ga]Ga-PSMA-617; according to the statistical analysis, its accumulation in the kidney one hour after administration was significantly lower (*p* < 0.05) than in the case of [^68^Ga]Ga-PSMA-617. We consider this as a success, as a similarly designed inhibitor based on these findings, which could reduce the renal burden for potential endoradiotherapy.

Accumulation of PSMA inhibitors in tumour was increasing with time, while they were progressively excreted by the urine.

The tumour/kidney ratio increased for the [^68^Ga]Ga-PSMA-617 inhibitor two hours after administration, which had a more favourable ratio than [^68^Ga]Ga-P19 two hours after administration (Fig. 5), but the difference was not significant. The other two inhibitors studied have low tumour/kidney ratios at both times, less than 0.5 (Fig. 5).

We would like to note as well that the reason why we are not able to visualize the prostate in any of the PET/CT images of the mice, which were all males, is that the mouse prostate does not produce PSMA in any quantity. In fact, only mammals other than humans which have expression of PSMA and are prone to have PCa are dogs (Coffey 2001).

## Conclusions

In this study, three novel PSMA inhibitors—P15, P16, and P19—were evaluated against the clinically established PSMA-617, with a focus on reversed synthetic strategy and linker region modifications. All compounds achieved high radiochemical purity and demonstrated good stability under physiological conditions. Inverse analogues [^68^Ga]Ga-P15 and [^68^Ga]Ga-P16 exhibited lower plasma protein binding compared to [^68^Ga]Ga-PSMA-617, however PET/CT imaging and biodistribution studies confirmed specific tumour targeting for all inhibitors. Our finding identified [^68^Ga]Ga-P19 with a diphenyl moiety in the linker region as a promising candidate for further development, including potential application in PSMA-based radiotheranostics using ¹⁷⁷Lu, a radionuclide with a longer half-life, with respect to longer time intervals from application, which are not possible using ^68^Ga.

## Supplementary Information

Below is the link to the electronic supplementary material.

Supplementary Material 1

## Data Availability

The datasets used and/or analysed during the current study are available from the corresponding authors on reasonable request.

## Abbreviations

^68^Ga: Gallium-68
ACN: Acetonitrile
AMBA: 4-(aminomethyl)benzoic acid
ATCC: American tissue culture collection
BSA: Bovine serum albumin
CHX: Cyclohexane
CT: Computed tomography
DOTA: 1,4,7,10-tetraazacyclododecane-1,4,7,10-tetraacetic acid
ESI-MS: Electrospray inonisation mass spectrometry
FOV: Field of view
Glu-urea-Glu: Glutamine-urea-glutamine
Glu-urea-Lys: Glutamine-urea-Lysine
HCL: Hydrochloric acid
HPLC: High performance liquid chromatography
ID/g: Injected dose per gram
LNCaP: Lymph node cancer of prostate
MALDI-MS: Matrix-assisted laser desorption/ionization mass spectrometry
MIP: Maximum intensity projection
PBS: Phosphate buffer
PCa: Prostate cancer
PET: Positron emission tomography
PSMA I&T: Prostatic Specific Membrane antigen Imaging and Therapy
R.O: Retro orbitally
ROI: Region of interest
SCID: Severe combined immunodeficiency
SPF: Specific pathogen free
TOF: Time of flight
TFA: Trifluoroacetic acid
