# Supplementary material for: Paving the way for future PSMA inhibitors: insights from comparative preclinical evaluations of structure modifications
Source: EJNMMI Radiopharm Chem. 2025 Sep 26;10:63. doi: 10.1186/s41181-025-00389-w (PMC12474757; doi:10.1186/s41181-025-00389-w)
Supplement: Supplementary file 1 — Supplementary Material 1 [file 41181_2025_389_MOESM1_ESM.docx]

**Paving the Way for Future PSMA Inhibitors: Insights from Comparative Preclinical Evaluations of Structure Modifications**

Katarína Hajduová^1^, Kateřina Dvořáková Bendová^1^, Miloš Petřík^1,2,3^, Martina Benešová-Schäfer^4^, Martin Schäfer^5^, Marián Hajdúch^1,2,3^, Zbyněk Nový^1,2^*****

1. Institute of Molecular and Translational Medicine, Faculty of Medicine and Dentistry, Palacky University, Olomouc, Czech Republic

2. Czech Advanced Technology and Research Institute, Palacky University, Olomouc, Czech Republic

3. Institute of Molecular and Translational Medicine, University Hospital, Olomouc, Czech Republic

4. Research Group Translational Radiotheranostics, German Cancer Research Center (DKFZ), Im Neuenheimer Feld 280, 69120 Heidelberg, Germany

5. Service Unit for Radiopharmaceuticals and Preclinical Studies, German Cancer Research Center (DKFZ), Im Neuenheimer Feld 280, 69120 Heidelberg, Germany

**E-Mail addresses**:

[katarina.hajduova01@upol.cz](mailto:katarina.hajduova01@upol.cz), [katerina.dvorakovabendova@upol.cz](mailto:katerina.dvorakovabendova@upol.cz), [milos.petrik@upol.cz](mailto:milos.petrik@upol.cz), [m.benesova@dkfz-heidelberg.de](mailto:m.benesova@dkfz-heidelberg.de), [martin.schaefer@dkfz-heidelberg.de](mailto:martin.schaefer@dkfz-heidelberg.de), [marian.hajduch@upol.cz](mailto:marian.hajduch@upol.cz), [zbynek.novy@upol.cz](mailto:zbynek.novy@upol.cz)

***Correspondence to**:

Zbynek Novy

Institute of Molecular and Translational Medicine

Faculty of Medicine and Dentistry

Palacky University Olomouc

Hnevotinska 1333/5

779 00 Olomouc

Czech Republic

E-mail: zbynek.novy@upol.cz

Phone: +420585632126

**Word count**: 2059

**Scheme S1**: General outline for PSMA-617 synthesis.

**Scheme S2**: General outline for P19 synthesis.

**Synthesis of the peptidomimetic glutamate-urea-lysine (Glu-urea-Lys) binding motif for PSMA-617 and P19**

5 g 2-CT resin (6.1 mmol, substitution capacity 1.22 mmol/g, 100–200 MESH) was first agitated in dry DCM for 45 min and then washed with dry DCM followed by reaction with 1.2 equiv Fmoc-Glu(O*t*Bu)-OH (**1**), and 4.8 equiv DIPEA in 150 mL of dry DCM. The coupling of the first protected amino acid on the resin proceeded over the course of 16 h with gentle agitation.

The glutamate-immobilized resin **2** was then washed with DCM with unreacted chlorotrityl groups remaining in the resin blocked with a mixture of DCM, MeOH, and DIPEA in a ratio of 17:2:1, respectively. Selective removal of the Fmoc-protecting group was realized by washing three times with 30 mL of a mixture of DMF and piperidine in a ratio of 1:1 in order to get product **3**.

In the next step, 16.3 mmol H-Lys(Fmoc)-O*t*Bu was dissolved in 100 mL of dry DMF and stirred with 16.3 mmol 1,1'-carbonyldiimidazol (CDI) and 16.6 mmol DIPEA at RT for 1 h. The glutamate-immobilized resin **3** was added afterward in one portion to the solution of the CDI-adduct of the lysine moiety and stirred for 16 h.

The product **4** was filtered off and washed with DCM and DMF. Selective removal of the Fmoc-protecting group was realized by washing with a mixture of DMF and piperidine in a ratio of 1:1 once for 2 min and then once again for 5 min. The resin-immobilized and *t*Bu-protected binding motif **5** was dried under vacuum and split into three portions.

**Linker coupling for PSMA-617 and P19**

Relative to the resin (0.1 mmol), 4 equiv Fmoc-2-Nal-L-Ala (Fmoc-2-naphthyl-L-alanine, 0.400 mmol) for PSMA-617 or 4 equiv Fmoc-3,3-dPh-L-Ala (Fmoc-3,3-diphenyl-L-alanine, 0.400 mmol) for P19 were activated with 3.92 equiv HBTU (0.392 mmol) in the presence of 4 equiv DIPEA (0.400 mmol) in 3 mL dry DMF. Two min after the addition of DIPEA, the solution was added to the DMF pre-swollen immobilized pharmacophore **5** and agitated at RT for 1 h. Selective removal of the Fmoc-protecting group from the products **6** and **11** was realized by washing with a mixture of DMF and piperidine in a ratio of 1:1 once for 2 min and then once again for 5 min in order to obtain the products **7** and **12**.

In the next step, for PSMA-617, 5 equiv Fmoc-TXA (trans-4-(Fmoc-aminomethyl)cyclohexanecarboxylic acid, 0.500 mmol) were reacted with 5 equiv Oxyma Pure (0.500 mmol) and 5 equiv DIC (1,1'-carbonyldiimidazol, 0.500 mmol) in 10 mL dry DMF:DCM in a ratio 1:1. The solution was added to the DMF pre-swollen immobilized pharmacophore **7** and agitated at RT for 1 h. Analogically, for P19, 4 equiv Fmoc-AMBA (4-(Fmoc-aminomethyl)benzoic acid, 0.400 mmol) were reacted with 3.92 equiv HBTU (0.392 mmol) and 4 equiv DIPEA (0.400 mmol) in 3 mL dry DMF. Two min after the addition of DIPEA, the solution was added to the DMF pre-swollen immobilized pharmacophore **12** and agitated at RT for 1 h. Selective removal of the Fmoc-protecting group from the products **8** and **13** was realized by washing with a mixture of DMF and piperidine in a ratio of 1:1 once for 2 min and then once again for 5 min in order to obtain the products **9** and **14**.

**Chelator conjugation for PSMA-617 and P19**

Relative to the resin (0.1 mmol), 4 equiv DOTA-*tris*(*t*Bu)ester (2-(4,7,10-tris(2-(t-butoxy)-2-oxoethyl)-1,4,7,10-tetraazacyclododecan-1-yl)acetic acid, 0.400 mmol) were activated with 3.95 equiv HBTU (0.395 mmol) in the presence of 4 equiv DIPEA (0.400 mmol) in 3 mL dry DMF. Two min after the addition of DIPEA, the solution was added to the DMF pre-swollen immobilized compounds **9** and **14** and agitated at RT for 3 h. The final products, i.e. **PSMA-617** and **P19**, were obtained by agitation and subsequent cleavage from the resin and *t*Bu deprotection within 3 h with a mixture consisting of trifluoroacetic acid (TFA), triisopropylsilane (TIPS), and water in a ratio of 95:2.5:2.5, respectively. The mixture of solvents from the product was evaporated and the crude compound was dissolved in acetonitrile (ACN) and water in a ratio of 1:1 for subsequent reversed-phase high-performance liquid chromatography (RP-HPLC) purification.

**Scheme S3**: General outline for P15 synthesis.

**Scheme S4**: General outline for P16 synthesis.

**Inverse synthesis of P15 and P16**

5 g 2-CT resin (6.6 mmol, substitution capacity 1.32 mmol/g, 100–200 MESH) was first agitated in dry DCM for 45 min and then washed with dry DCM followed by reaction with 8 equiv *trans*-1,4-diamino-cyclohexan (**1**), and 10 mL DIPEA in 50 mL of dry DCM. The coupling of the first building block on the resin proceeded over the course of 16 h with gentle agitation.

The *trans*-1,4-diamino-cyclohexan-immobilized resin **2** was filtered off and washed with DCM, 250 mL tetrahydrofuran (THF) and 250 mL THF/water in a ratio of 3:1.

Relative to the resin (0.65 mmol), 3.3 equiv Fmoc-2-Nal-D-Ala (Fmoc-2-naphthyl-D-alanine, 1.95 mmol) for P15 or Fmoc-2-Nal-L-Ala (Fmoc-2-naphthyl-L-alanine, 1.95 mmol) for P16 were activated with 3.3 equiv HBTU (1.95 mmol) in the presence of 3.3 equiv DIPEA (1.95 mmol) in 3 mL dry DMF. Two min after the addition of DIPEA, the solution was added to the DMF pre-swollen immobilized compound **2** and agitated at RT for 1 h. Selective removal of the Fmoc-protecting group from the product **3** was realized by washing with a mixture of DMF and piperidine in a ratio of 1:1 once for 2 min and then once again for 5 min in order to obtain the product **4**.

In the next step, 3.3 equiv Fmoc-Glu-O*t*Bu (Fmoc-L-glutamate-O*t*Bu, 1.95 mmol) were activated with 3.3 equiv HBTU (1.95 mmol) in the presence of 3.3 equiv DIPEA (1.95 mmol) in 3 mL dry DMF. Two min after the addition of DIPEA, the solution was added to the DMF pre-swollen immobilized compound **4** and agitated at RT for 1 h. Selective removal of the Fmoc-protecting group from the product **5** was realized by washing with a mixture of DMF and piperidine in a ratio of 1:1 once for 2 min and then once again for 5 min in order to obtain the product **6**.

Further conjugation step involved 10.0 mmol H-Glu(OtBu)-OtBu (H-glutamate-(OtBu)-OtBu) which was dissolved in 10 mL of dry DMF and stirred with 10.0 mmol 1,1'-carbonyldiimidazol (CDI) and 10.0 mmol DIPEA at RT for 3 h. The resin-immobilized resin **6** was added afterward in one portion to the solution of the CDI-adduct of the glutamate moiety and stirred for 16 h yielding in compound **7**.

Subsequently, compound **8** was obtained by agitation and subsequent cleavage from the resin and *t*Bu deprotection within 3 h with a mixture consisting of TFA, TIPS, and water in a ratio of 95:2.5:2.5, respectively. The mixture of solvents from the product was evaporated and the crude compound was dissolved in acetonitrile (ACN) and water in a ratio of 1:1 for subsequent RP-HPLC purification.

Finally, for 30 mg of purified lyophilised substance **8**, 1.5 equiv DOTA-NHS ester were diluted in 0.3 mL dry DMF and 1.5 equiv DIPEA were added. The solution was added to the compound **8** and agitated at RT for 16 h. The final products, i.e. **P15** and **P16**, were upon the final RP-HPLC purification.

**Quality control (ESI-MS)**

**Figure S1**: Low resolution ESI-MS of P15. The calculated *m*/*z* for [M + H^+^]^+^ is 1000.46 g/mol.

**Figure S2**: Low resolution ESI-MS of P16. The calculated *m*/*z* for [M + H^+^]^+^ is 1000.46 g/mol.

**Figure S3**: Low resolution ESI-MS of P19. The calculated *m*/*z* for [M + H^+^]^+^ is 1062.47 g/mol.

**Quality control (MALDI-MS)**

**Figure S4**: High resolution MALDI-MS of P15. The calculated *m*/*z* for [M + H^+^]^+^ is 1000.4583 g/mol.

**Figure S5**: High resolution MALDI-MS of P16. The calculated *m*/*z* for [M + H^+^]^+^ is 1000.4583 g/mol.

**Figure S6**: High resolution MALDI-MS of P19. The calculated *m*/*z* for [M + H^+^]^+^ is 1062.4739 g/mol.

**Quality control (HPLC)**

**Figure S7**: HPLC of P15.

**Figure S8**: HPLC of P16.

**Figure S9**: HPLC of P19.

Table S1: Optimization of labelling conditions for PSMA inhibitors using 68Ga investigated parameter is the labelling time.

|  | **Amount**    **(µg)** | **Temperature (°C)** | **Time (min)** | **Radiochemical yield (%)** |
| --- | --- | --- | --- | --- |
|  | 5 | 95 | 1 | 94,53 |
| **[^68^Ga]Ga PSMA-617** | 5 | 95 | 5 | 99,32 |
|  | 5 | 95 | 10 | 99,36 |
|  | 5 | 95 | 1 | 96,79 |
| **[^68^Ga]Ga P15** | 5 | 95 | 5 | 99,19 |
|  | 5 | 95 | 10 | 99,30 |
|  | 5 | 95 | 1 | 97,63 |
| **[^68^Ga]Ga P16** | 5 | 95 | 5 | 99,45 |
|  | 5 | 95 | 10 | 99,80 |
|  | 5 | 95 | 1 | 95,36 |
| **[^68^Ga]Ga P19** | 5 | 95 | 5 | 99,38 |
|  | 5 | 95 | 10 | 99,75 |

Table S2: Optimization of labelling conditions for PSMA inhibitors using 68Ga investigated parameter is the amount of PSMA inhibitor.

|  | **Amount**    **(µg)** | **Temperature (°C)** | **Time (min)** | **Radiochemical yield (%)** |
| --- | --- | --- | --- | --- |
|  | 0,5 | 95 | 5 | 98,36 |
| **[^68^Ga]Ga PSMA-617** | 1 | 95 | 5 | 98,83 |
|  | 5 | 95 | 5 | 99,32 |
|  | 0,5 | 95 | 5 | 98,22 |
| **[^68^Ga]Ga P15** | 1 | 95 | 5 | 98,49 |
|  | 5 | 95 | 5 | 99,19 |
|  | 0,5 | 95 | 5 | 98,78 |
| **[^68^Ga]Ga P16** | 1 | 95 | 5 | 98,92 |
|  | 5 | 95 | 5 | 99,45 |
|  | 0,5 | 95 | 5 | 97,90 |
| **[^68^Ga]Ga P19** | 1 | 95 | 5 | 98,46 |
|  | 5 | 95 | 5 | 99,38 |

Table S3: Biodistribution data of [^68^Ga]Ga-PSMA-617, -P15, -P16 and -P19 inhibitors in LNCaP tumour bearing mice at 1 h and 2 h p.i. Values are shown as the mean ± standard deviations (n=4).

| **[^68^Ga]Ga**  **PSMA inhibitors** | **[^68^Ga]Ga PSMA-617** | | **[^68^Ga]Ga P15** | | **[^68^Ga]Ga P16** | | | **[^68^Ga]Ga P19** | |
| --- | --- | --- | --- | --- | --- | --- | --- | --- | --- |
|  | **1 h p.i.** | **2 h p.i.** | **1 h p.i.** | **2 h p.i.** | **1 h p.i.** | | **2 h p.i.** | **1 h p.i.** | **2 h p.i.** |
|  | **MV ± SD** | **MV ± SD** | **MV ± SD** | **MV ± SD** | **MV ± SD** | **MV ± SD** | | **MV ± SD** | **MV ± SD** |
| **blood** | 1.02 ± 0.30 | 0.56 ± 0.15 | 0.71 ± 0.07 | 0.64 ± 0.13 | 0.19 ± 0.06 | 0.08 ± 0.01 | | 0.41 ± 0.07 | 0.23 ± 0.06 |
| **spleen** | 4.23 ± 0.15 | 6.85 ± 3.43 | 1.03 ± 0.34 | 2.69 ± 1.10 | 7.72 ± 4.23 | 9.68 ± 4.07 | | 8.44 ± 2.21 | 5.10 ± 0.77 |
| **pancreas** | 0.26 ± 0.06 | 0.16 ± 0.02 | 0.16 ± 0.03 | 0.16 ± 0.03 | 0.10 ± 0.03 | 0.04 ± 0.02 | | 0.16 ± 0.03 | 0.07 ± 0.01 |
| **stomach** | 0.94 ± 1.23 | 0.18 ± 0.05 | 0.34 ± 0.33 | 0.27 ± 0.14 | 0.74 ± 1.10 | 0.16 ± 0.09 | | 0.29 ± 0.11 | 1.12 ± 1.11 |
| **intestine** | 1.18 ± 1.44 | 0.50 ± 0.15 | 0.30 ± 0.14 | 0.48 ± 0.11 | 0.68 ± 0.97 | 0.44 ± 0.15 | | 0.57 ± 0.38 | 2.12 ± 1.30 |
| **kidneys** | 50.71 ± 4.52 | 6.36 ± 3.28 | 2.67 ± 1.32 | 1.45 ± 0.38 | 6.52 ± 1.81 | 1.62 ± 0.57 | | 4.74 ± 1.63 | 2.35 ± 0.53 |
| **liver** | 2.64 ± 0.66 | 3.23 ± 1.20 | 1.27 ± 0.70 | 2.18 ± 0.48 | 4.02 ± 0.78 | 4.35 ± 1.19 | | 3.20 ± 1.10 | 3.05 ± 0.84 |
| **heart** | 0.41 ± 0.11 | 0.31 ± 0.03 | 0.25 ± 0.02 | 0.23 ± 0.06 | 0.17 ± 0.02 | 0.12 ± 0.03 | | 0.24 ± 0.04 | 0.12 ± 0.03 |
| **lung** | 1.18 ± 0.29 | 0.92 ± 0.21 | 0.59 ± 0.15 | 0.39 ± 0.05 | 0.64 ± 0.15 | 0.37 ± 0.07 | | 0.89 ± 0.17 | 0.51 ± 0.08 |
| **muscle** | 0.14 ± 0.05 | 2.01 ± 0.51 | 0.12 ± 0.03 | 0.08 ± 0.01 | 0.05 ± 0.01 | 0.05 ± 0.04 | | 0.22 ± 0.10 | 0.04 ± 0.01 |
| **femur** | 0.32 ± 0.07 | 0.89 ± 0.25 | 0.26 ± 0.07 | 0.31 ± 0.09 | 0.38 ± 0.15 | 0.36 ± 0.15 | | 0.92 ± 0.57 | 0.37 ± 0.16 |
| **tumour** | 5.25 ± 1.76 | 7.07 ± 2.68 | 0.56 ± 0.22 | 0.52 ± 0.28 | 0.75 ± 0.10 | 0.61 ± 0.14 | | 3.52 ± 0.36 | 2.00 ± 0.47 |

**Figure S10**: Radio-HPLC of **[^68^Ga]Ga P15**

**Figure S11**: Radio-HPLC of **[^68^Ga]Ga P16**

**Figure S12**: Radio-HPLC of **[^68^Ga]Ga P19**

**Figure S13**: Radio-HPLC of **[^68^Ga]Ga PSMA-617**
